# Relationship between elevated serum direct bilirubin and atrial fibrillation risk among patients with coronary artery disease

**DOI:** 10.3389/fmed.2025.1405682

**Published:** 2025-02-14

**Authors:** Yanbin Song, Wenhua Li

**Affiliations:** ^1^Department of Cardiology, Wujin Hospital Affiliated With Jiangsu University, Changzhou, China; ^2^Department of Cardiology, The Wujin Clinical College of Xuzhou Medical University, Changzhou, China

**Keywords:** direct bilirubin, atrial fibrillation, coronary artery disease, risk, total cholesterol

## Abstract

**Background:**

Observational studies have shown that the direct bilirubin (DBIL) is correlated with metabolic syndrome and cardiovascular disease. However, it remains unclear whether DBIL is associated with atrial fibrillation (AF) risk in the patients with coronary artery disease (CAD). This study aimed to investigate the association between serum DBIL levels and AF in CAD patients.

**Methods:**

A total of 937 patients diagnosed with CAD were retrospectively included. Serum total bilirubin (TBIL), DBIL, lipid profiles, and other data were collected and analyzed between the AF and non-AF groups. The characteristics of participants were compared based on their DBIL tertiles. Univariate and multivariate logistic regression models, as well as restricted cubic spline (RCS) regression, were used to explore the relationship between DBIL and AF.

**Results:**

AF was observed in 72 (7.7%) patients. There was a significant higher level of DBIL in the AF patients compared to non-AF patients (*p* < 0.001). Individuals from the DBIL T3 group, when compared to those from the T1 or T2 groups, were more likely to have a higher proportion of AF and lower levels of total cholesterol (TC), triglycerides (TG), low-density lipoprotein cholesterol (LDL-C), apolipoprotein B (Apo B) and triglyceride-glucose (TyG) (all *p* < 0.001). Univariate logistic regression showed that the OR for AF in patients in T3 was 2.796 (95% CI, 1.528–5.116, *p* = 0.001) compared with participants in T1. The result remained consistent in the multivariate logistic regression (T3 versus 1: adjusted OR: 2.239). The RCS curve demonstrated a significant nonlinear association between DBIL and AF. Subgroup analysis revealed that this association was significant among patients aged ≥65 years old, with body mass index (BMI) < 25, and with diabetes mellitus (DM).

**Conclusion:**

The study suggested a robust relationship between higher levels of DBIL and an increased risk of AF in CAD patients. The association of elevated DBIL with the incidence of AF was higher in CAD patients older than 65 years, with a BMI < 25, and those with DM.

## Introduction

Atrial fibrillation (AF) is the most frequent cardiac arrhythmia, and is associated with significant health and economic burden on patients worldwide (1). However, the preventive measurement for AF is not satisfied so date due to the unclear underling mechanism (2, 3).

As we all know, AF and coronary artery disease (CAD) often co-exist based on multiple similar risk factors. The prevalence of CAD in patients with AF is estimated to range from 17 to 46.5% (4, 5). Risk factors for CAD such as elderly age and diabetes mellitus (DM) usually result in atrial remodeling, which includes structural and electrical transformation that contribute to the incidence and development of AF (6–8). Therefore, it is critical to identify and control these risk factors for the prevention and treatment of AF in individuals with CAD.

Some correlative processes including inflammation, endothelial dysfunction and oxidative stress have been demonstrated to be associated with cardiovascular disease in basic and clinical research (9–11). Bilirubin, a tetrapyrrolic compound, can be oxidized by H2O2 during inflammatory to form several degradation products that may contribute to the pathogenesis of certain diseases (12). The direct bilirubin (DBIL) is considered an inflammatory marker and has been proved to play an important role in predicting the poor prognosis of patients with acute and chronic heart failure (13, 14). Moreover, DBIL has been identified as an independent risk factor for new-onset postoperative atrial fibrillation (POAF) after cardiac surgery (15). A retrospective cohort study conducted on thyrotoxic patients receiving radioactive iodine therapy revealed that DBIL was an important risk factor for predicting AF (16). However, Weiping Sun (17) found that DBIL was not an independent predictive factors for the occurrence of paroxysmal atrial fibrillation (pAF). In addition, a case–control study reported significantly lower levels of DBIL in AF patients compared to healthy individuals (18).

Previous studies on the association between serum DBIL and AF risk have yielded inconsistent results. The epidemiological evidence regarding the association of DBIL with risk of AF among patients with CAD remains unclear. Therefore, our study aims to determine whether an elevated serum DBIL concentration is associated with a higher risk of AF in a group of CAD patients.

## Patients and methods

### Study patients

987 consecutive patients aged ≥18 years old were retrospectively enrolled in Wujin Hospital Affiliated with Jiangsu University. All the patients were diagnosed with CAD for the first time through coronary angiography (CAG) between January 2019 and December 2021. CAD was defined as a stenosis of 50% or more in the diameter of the major coronary blood vessels.

The patients with chronic hepatitis/cirrhosis, hemolytic anaemia, pregnancy, intoxication, biliary obstruction disease, systemic inflammation, cancer or missing essential data for total bilirubin (TBIL) or DBIL, lipid profile, and ongoing lipid-lowering therapy were excluded. Finally, a total of 937 eligible patients were included and analyzed in the current study.

The study was conducted in compliance with the principles outlined in the Declaration of Helsinki (as revised in 2013). Ethics approval was obtained by the Ethics Committee of Wujin Hospital Affiliated with Jiangsu University, China (2023-SR-055). Since the data was retrospectively collected, written informed consent from the study participants had not been obtained.

### Measurement of covariates

Clinical characteristics and demographic parameters at baseline, including age, sex, BMI, history of hypertension, DM, smoking, and drinking status were obtained. Venous blood samples were collected after at least 8 h of fasting. Biochemical parameters such as TBIL, DBIL, hemoglobin, alanine aminotransferase (ALT), aspartate aminotransferase (AST), urea nitrogen (BUN), creatinine (Cr), uric acid (UA), fasting blood glucose (FBG), lipid profiles including total cholesterol (TC), triglycerides (TG), high-density lipoprotein cholesterol (HDL-C), low-density lipoprotein cholesterol (LDL-C), apolipoprotein A-1 (Apo A-1) and apolipoprotein B (Apo B) were measured using standardized laboratory methods. The BMI was calculated using the formula: [weight/(Height*Height)] (kg/m2). The TyG (triglyceride-glucose) index values were evaluated as follows: ln [fasting TG (mg/dL) × FPG (mg/dL)/2].

The presence of AF (including paroxysmal AF, persistent AF and permanent AF) was confirmed using a 12-lead electrocardiogram (ECG) or a 24-h Holter monitor. AF rhythm was defined as (I) irregular R-R intervals (II) absence of distinct repeating P waves (III) irregular atrial activity show on ECG (2). The ECG results were confirmed by both researchers.

The severity of coronary artery stenosis was evaluated using the Gensini score (GS) based on the results of CAG for included cases. And the number of diseased coronary vessels with ≥50% stenosis was calculated in patients according to the selective coronary angiography. Patients were assigned into single-vessel CAD (1 diseased vessel) and multi-vessel CAD (≥2 diseased vessels).

### Statistical analysis

Normally continuous variables were presented as means ± standard deviation, while categorical variables were presented as numbers and percentages. Non-normally distributed variables were expressed as median [interquartile rang] (IQR). The *t*-student test, One-way ANOVA, Kruskal-Wallis H test and the chi-square test were used to determine significant differences among groups as appropriate. Univariate and multivariate logistic regression analyses were performed to assess the association of DBIL with AF. A curve of RCS regression model was conducted to explore the potential nonlinear association between DBIL and AF. Subgroup analyses on the association of DBIL with AF based on age, gender, BMI, and DM were carried out. The results were reported as odds ratio (OR) with 95% confidence intervals (95% CI). A two-tailed *p* value less than 0.05 was considered a threshold for significance. Data analysis was performed using IBM SPSS version 25.

## Results

### Patients characteristics

**Figure 1:**
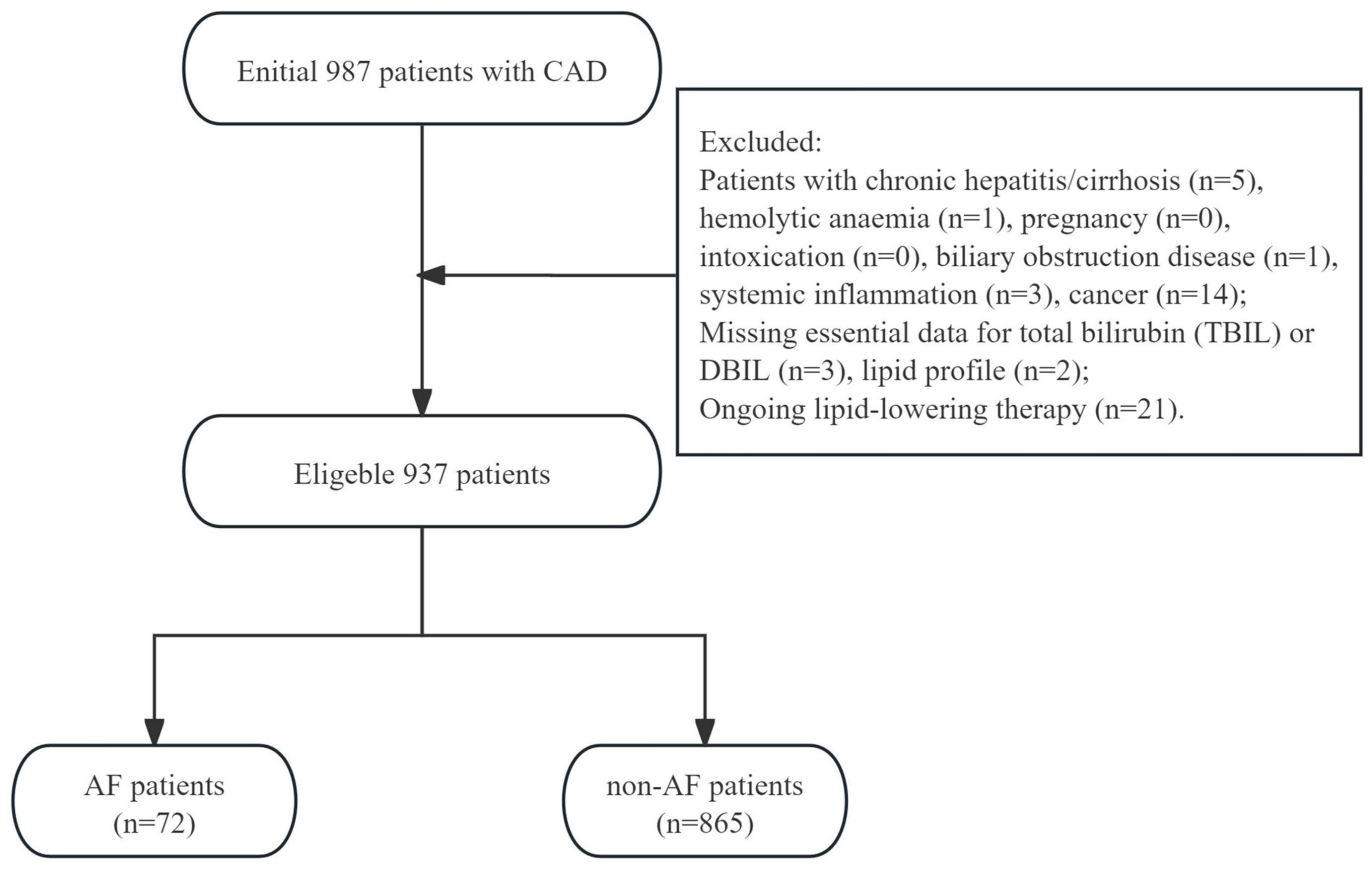
The flow-chart of study patients.

The flow diagram of patient selection is presented in Figure 1. This study analyzed 937 patients with CAD. The demographic, clinical, and laboratory characteristics of the study patients are shown in Table 1. The mean age of all patients was 67 (57,73) years, including 643 (86.7%) males. A total of 72 (7.7%) AF patients were observed. All patients were grouped according to the presence of AF.

**Table 1:** Baseline characteristics of the study patients.

Variables	Total (*n* = 937)	AF group (*n* = 72)	Non-AF group (*n* = 865)	*p* value
Age, median (IQR),y	67 (57, 73)	74 (68, 79)	66 (57, 73)	<0.001
Male (*n*, %)	643 (68.6)	39 (54.2)	604 (69.8)	0.006
Hypertension (*n*, %)	696 (74.3)	58 (80.6)	638 (73.8)	0.205
DM (*n*, %)	320 (34.1)	29 (40.3)	291 (33.6)	0.254
Smoking (*n*, %)	449 (47.9)	27 (37.5)	422 (48.8)	0.065
Drinking (*n*, %)	223 (23.8)	16 (22.2)	207 (23.9)	0.744
Gensini score	38.00 (20.00, 63.50)	25.25 (12.00, 52.88)	40.00 (21.00, 64.00)	0.010
Multi-vessel (*n*, %)	531 (56.7)	33 (45.8)	498 (57.6)	0.053
BMI, median (IQR), kg/m2	24.46 (22.23, 26.70)	24.62 (22.49, 27.75)	24.45 (22.22, 26.67)	0.549
Hemoglobin, g/L	137.84 ± 16.14	136.11 ± 15.27	137.98 ± 16.21	0.345
TBIL, median (IQR), μmol/L	14.80 (11.50, 19.20)	16.30 (13.40, 23.28)	14.60 (11.35, 19.00)	0.003
DBIL, median (IQR)μmol/L	2.70 (2.00, 3.60)	3.35 (2.40, 4.95)	2.60 (2.00, 3.60)	<0.001
ALT, U/L	21 (15, 34)	18.50 (12.25, 45.50)	21 (15, 33)	0.595
AST, U/L	26 (19.72)	24 (18, 124.75)	26 (19, 69.5)	0.592
BUN, mmol/L	5.49 (4.58, 6.65)	6.02 (4.48, 7.78)	5.45 (4.58, 6.60)	0.082
Cr, μmol/l	71.10 (61.40, 82.00)	73.45 (62.25, 90.63)	70.90 (61.35, 81.30)	0.120
UA, μmol/l	342.40 (286.40, 407.05)	349.10 (290.55, 434.10)	341.20 (284.55, 406.60)	0.532
FBG, mmol/L,	5.51 (4.88, 6.94)	5.56 (4.92, 6.87)	5.51 (4.88, 6.94)	0.837
TC, median (IQR), mmol/L	4.47 (3.80, 5.21)	3.87 (3.31, 4.75)	4.50 (3.83, 5.25)	<0.001
TG, median (IQR, mmol/L)	1.58 (1.15, 2.29)	1.46 (1.07, 2.08)	1.58 (1.16, 2.32)	0.161
HDL-C, median (IQR), mmol/L	1.12 (0.95, 1.32)	1.05 (0.93, 1.37)	1.13 (0.96, 1.32)	0.342
LDL-C, median (IQR), mmol/L	3.01 (2.47, 3.57)	2.55 (2.07, 3.23)	3.04 (2.51, 3.59)	<0.001
ApoA-1, median (IQR), g/L	1.17 (1.03, 1.33)	1.12 (1.00, 1.35)	1.17 (1.04, 1.33)	0.092
Apo B, median (IQR), g/L	0.88 (0.73, 1.04)	0.75 (0.60, 0.98)	0.89 (0.74, 1.04)	<0.001
TyG	1.60 ± 0.67	1.48 ± 0.60	1.61 ± 0.67	0.097

The AF patients were notably older than non-AF patients and had a significantly lower proportion of male gender (all *p* < 0.01). Besides this, the patients with AF had significantly higher levels of TBIL and DBIL compared to those with non-AF (*p* = 0.003 and *p* < 0.001, respectively). While, significant lower concentrations of TC, LDL-C and ApoB levels were observed in the AF group (*p* < 0.001). However, there were no statistical differences neither in percentage of hypertension, DM, smoking, drinking, nor in levels of BMI, hemoglobin, ALT, AST, BUN, Cr, UA, FBG, TG, HDL-C, ApoA1 or TyG index between the two groups (*p*-values >0.05).

### Characteristics of participants by the DBIL tertiles

The 937 CAD participants were assigned into three groups based on their DBIL levels in tertiles: T1 (≤2.2, *n* = 328), T2 (>2.2 and ≤ 3.2, *n* = 298), and T3 (>3.2, *n* = 311). The characteristics of the patients according to their DBIL levels were summarized in Table 2.

**Table 2:** Characteristics of study participants by the DBIL tertiles.

Variables	T1 (*n* = 328)	T2 (*n* = 298)	T3 (*n* = 311)	*p* value
Age (IQR, years)	66 (57, 72)	66.5 (57, 74)	67 (58, 74)	0.177
Male (*n*, %)	187 (57)	209 (70.1) ^*^	247 (79.4) ^## ^^	<0.001
Hypertension (*n*, %)	247 (75.3)	220 (73.8)	229 (73.6)	0.869
DM (*n*, %)	121 (36.9)	95 (31.9)	104 (33.4)	0.397
AF (*n*, %)	16 (4.9)	17 (5.7)	39 (12.5) ^# ^^	<0.001
Smoking (*n*, %)	136 (41.5)	149 (50) ^*^	164 (52.7) ^#^	0.012
Drinking (*n*, %)	63 (19.2)	71 (23.8)	89 (28.6) ^#^	0.020
Gensini score	38.50 (20.50, 61.00)	34.75 (18.38, 60.63)	41.00 (21.00, 74.00)	0.198
Multi-vessel (*n*, %)	194 (59.1)	170 (57.0)	167 (53.7)	0.376
Hemoglobin, g/L	135.28 ± 16.35	138.37 ± 15.62 ^*^	140.03 ± 16.10 ^##^	0.001
BMI (IQR, kg/m^2^)	24.39 (22.58, 26.83)	24.23 (22.06, 26.50)	24.57 (21.97, 26.78)	0.547
TBIL (IQR, μmol/L)	10.55 (9.10, 12.48)	14.80 (13.20, 17.00) ^**^	21.30 (17.89, 25.90) ^##^^^	<0.001
DBIL (IQR, μmol/L)	1.80 (1.50, 2.10)	2.70 (2.50, 2.93) ^**^	4.10 (3.60, 5.00) ^## ^^^	<0.001
ALT, U/L	19 (13, 30)	21 (15, 32.25)	24 (17, 40) ^## ^^^	<0.001
AST, U/L	23 (18, 41)	26 (19, 59.25) ^*^	35 (21, 130) ^## ^^^	<0.001
BUN, mmol/L	5.70 (4.75, 6.73)	5.50 (4.64, 6.66)	5.33 (4.46, 6.53)	0.059
Cr, μmol/l	69.2 (60.3, 78.0)	72.55 (61.98, 84.23) ^*^	72.1 (61.5, 85.4) ^#^	0.002
UA, μmol/l	343.2 (285.85, 405.)	342.85 (290, 404.53)	341.2 (79.89, 410.6)	0.998
FBG, mmol/L,	5.57 (4.86, 6.98)	5.37 (4.86, 6.73)	5.65 (4.92, 7.02)	0.133
TC (IQR, mmol/L)	4.75 (4.16, 5.56)	4.51 (3.90, 5.21) ^**^	4.05 (3.51, 4.79) ^## ^^^	<0.001
TG (IQR, mmol/L)	1.90 (1.31, 2.71)	1.59 (1.16, 2.21) ^**^	1.40 (1.03, 1.87) ^## ^^^	<0.001
HDL-C (IQR, mmol/L)	1.13 (0.95, 1.32)	1.11 (0.96, 1.27)	1.13 (0.95, 1.36)	0.467
LDL-C (IQR, mmol/L)	3.28 (2.78, 3.88)	3.05 (2.55, 3.54) ^**^	2.66 (2.26, 3.24) ^## ^^^	<0.001
Apo A-1 (IQR, g/L)	1.18 (1.04, 1.32)	1.15 (1.04, 1.32)	1.17 (1.01, 1.37)	0.999
Apo B (IQR, g/L)	0.96 (0.82, 1.12)	0.91 (0.75, 1.04) ^**^	0.79 (0.66, 0.94) ^## ^^^	<0.001
TyG	1.76 ± 0.67	1.57 ± 0.63 ^**^	1.47 ± 0.66 ^##^	<0.001

^*^p < 0.05 compared to T1; ^**^p < 0.001 compared to T1; ^#^*p* < 0.05 compared to T1; ^##^*p* < 0.001 compared to T1; ^^^*p* < 0.05 compared to T2; ^^^^*p* < 0.001 compared to T2.

The patients in the T3 group seemed not to be older than those in the T1 and T2 groups (*p* = 0.177). The T3 exhibited a higher percentage of male gender (T1 vs. T2 vs. T3: 57% vs. 70.1% vs. 79.4%, *p* < 0.001). The proportion of AF was significantly higher in the T3 group compared to the T1 or T2 groups (T1 vs. T2 vs. T3: 4.9% vs. 5.7% vs. 12.5%, *p* < 0.001). And more smoker and drinker were observed in the T3 group (all *p* < 0.05).

Patients from the T3 group were more likely to have higher levels of TBIL, DBIL, ALT and AST compared to those from the T1 or T2 groups, and they had lower levels of TC, TG, LDL-C, ApoB, and TyG (all *p* < 0.001). Additionally, patients in the DBIL T3 or T2 groups had higher concentrations of hemoglobin in contrast to those in the T1 (*p* = 0.001). However, there were no significant differences in the proportions of hypertension, DM and multi-vessel, or in the levels of BMI, Gensini score, HDL-C and ApoA1.

### Logistic regression analysis of variables with AF

The binary logistic regression was conducted to establish the association between DBIL and AF in patients with CAD. The results of univariate and multivariate logistic regression analyses for the relationship between variables and AF are presented in Table 3.

**Table 3:** Univariate and multivariate analysis of variables associated with AF in patients with CAD.

Variables	Univariate analysis		Multivariate analysis	
	OR (95% CI)	*p* value	OR (95% CI)	*p* value
Age	1.084 (1.054–1.114)	<0.001	1.072 (1.042–1.104)	<0.001
Male	0.511 (0.314–0.830)	0.007	0.564 (0.329–0.967)	0.038
Smoking	0.630 (0.384–1.034)	0.067		0.243
Drinking	0.908 (0.510–1.617)	0.744		0.511
Hypertension	1.474 (0.807–2.694)	0.207		0.378
DM	1.330 (0.814–2.175)	0.255		0.269
Multi-vessel	0.624 (0.385–1.011)	0.055		
Gensini score	0.991 (0.983–0.999)	0.035	0.991 (0.983–1.000)	0.047
Hemoglobin	0.993 (0.978–1.008)	0.344		
BMI	1.038 (0.974–1.106)	0.254		
ALT	1.000 (0.994–1.006)	0.949		
AST	1.000 (0.999–1.002)	0.767		
BUN	1.000 (0.989–1.011)	0.933		
Cr	1.013 (1.003–1.023)	0.013		0.176
UA	1.001 (0.999–1.003)	0.309		
FBG	0.977 (0.875–1.090)	0.673		
TC	0.544 (0.417–0.709)	<0.001		0.248
TG	0.760 (0.582–0.992)	0.044		0.622
HDL-C	0.857 (0.367–2.001)	0.721		
LDL-C	0.460 (0.331–0.640)	<0.001	0.588 (0.414–0.833)	0.003
Apo A-1	0.346 (0.114–1.049)	0.061		
Apo B	0.081 (0.026–0.252)	<0.001		0.880
TyG	0.723 (0.493–1.060)	0.097		
DBIL T1	Reference		Reference	
T2	1.180 (0.585–2.379)	0.644	1.010 (0.488–2.090)	0.979
T3	2.796 (1.528–5.116)	0.001	2.239 (1.152–4.351)	0.017

The univariate regression analysis showed that the elevated DBIL was significantly associated with AF risk (T3 versus 1, OR = 2.796, 95% CI: 1.528–5.116, *p* = 0.001). Additionally, older age (OR: 1.084, 95% CI: 1.054–1.114, *p* < 0.001) and Cr (OR = 1.013, 95%CI: 1.003–1.023) were positively associated with AF risk. Male gender (OR: 0.511, 95%CI: 0.314–0.830, *p* = 0.007), TC (OR: 0.544, 95% CI: 0.417–0.709, *p* < 0.001), TG (OR: 0.760, 95% CI: 0.582–0.992, *p* = 0.044), LDL-C (OR: 0.460, 95% CI: 0.331–0.640, *p* < 0.001), Apo B (OR: 0.081, 95% CI: 0.026–0.251, *p* < 0.001) were significantly associated with a low risk of AF. No significant association were observed between smoking, drinking, hypertension, DM, BMI, hemoglobin, ALT, AST, BUN, UA, FBG, HDL-C, Apo A1, or TyG and the risk of AF.

The variables that had a *p*-value <0.05 in the univariate analyses and traditional risk factors, including smoking, drinking, hypertension and DM, were used in the multivariate logistic regression analysis model. After adjustment for other confounding factors, the OR of AF for patients in T3 was 2.239 (95% CI: 1.152–4.351) compared with participants in T1 (T3 versus 1, *p* = 0.017). In addition, older age (OR: 1.073, 95% CI: 1.042–1.104, *p* < 0.001) was an independent predictive for AF. Furthermore, the analysis revealed that LDL (OR: 0.588, 95% CI: 0.414–0.833, *p =* 0.001) remained inversely associated with the risk of AF. Moreover, the male gender (OR: 0.564, 95% CI: 0.309–0.918, *p* = 0.038) was found to be negatively correlated with AF risk.

**Figure 2:**
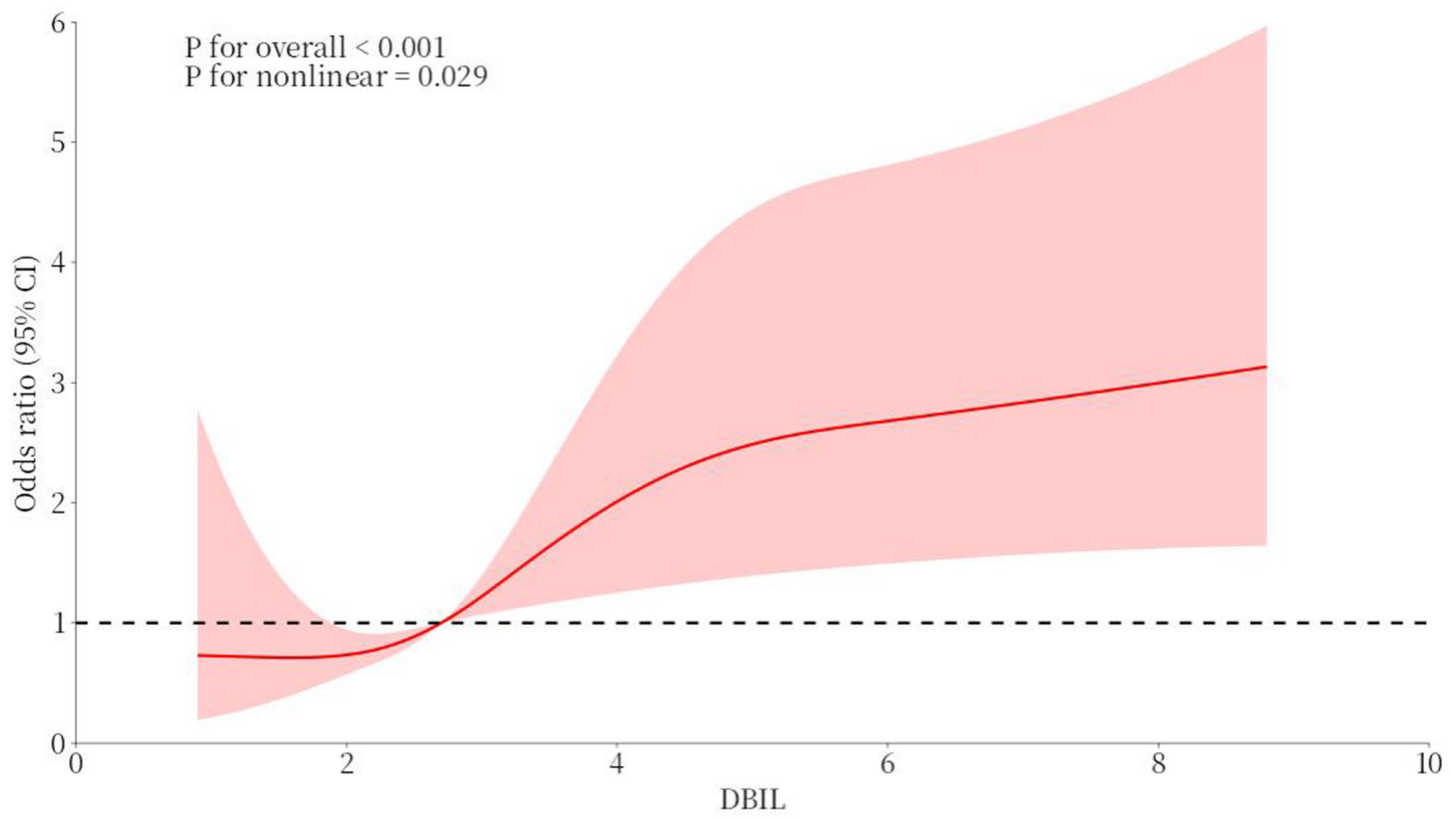
The RCS curve of the association between DBIL and AF among CAD patients.

Furthermore, a RCS curve of the DBIL for the prediction of AF among CAD patients had been displayed in Figure 2. The RCS regression model indicated that a significant nonlinear relationship between RAR and incident AF (*p* for overall < 0.001, *p* for nonlinear = 0.029). The inflection point was found at 2.7 for RAR.

### Subgroup stratification analysis for the relationship between DBIL and AF

Moreover, stratified analyses of the relationship between DBIL and AF risk based on age, gender, BMI, and DM were presented in Table 4. The association between higher DBIL and the risk of AF was stronger in CAD patients older than 65 years (OR: 3.255, 95% CI 1.466–7.226, *p* = 0.004). Higher DBIL levels were associated with AF in CAD patients with a BMI <25 (OR: 3.278, 95% CI 1.223–8.788, *p* = 0.018). The OR for AF in patients with DM in T3 was 3.540 (95% CI: 1.162–10.786) compared to participants in T1 (T3 versus 1, *p* = 0.026).

**Table 4:** Stratified analyses of the association between DBIL and AF in CAD patients.

Subgroup	Adjusted OR (95% CI)	*p* value
Age		
<65		
DBIL T1	Reference	
T2	0.503 (0.120–2.109)	0.348
T3	0.844 (0.229–3.118)	0.799
≥65		
DBIL T1	Reference	
T2	1.357 (0.569–3.237)	0.491
T3	3.255 (1.466–7.226)	0.004
Gender		
Male		
DBIL T1	Reference	
T2	1.027 (0.346–3.055)	0.961
T3	2.332 (0.906–6.003)	0.079
Female		
DBIL T1	Reference	
T2	0.948 (0.334–2.689)	0.920
T3	1.978 (0.716–5.463)	0.188
BMI		
<25		
DBIL T1	Reference	
T2	1.052 (0.346–3.200)	0.929
T3	3.278 (1.223–8.788)	0.018
≥25		
DBIL T1	Reference	
T2	1.2670 (0.465–3.416)	0.650
T3	1.478 (0.555–3.940)	0.429
DM		
Yes		
DBIL T1	Reference	
T2	2.442 (0.796–7.488)	0.118
T3	3.540 (1.162–10.786)	0.026
No		
DBIL T1	Reference	
T2	0.556 (0.203–1.519)	0.252
T3	1.775 (0.793–3.969)	0.163

## Discussion

In the study, there were two key findings. Firstly, the results suggested a significant relationship between elevated DBIL and AF risk in patients with CAD. Secondly, the correlation of elevated DBIL with the risk of AF in CAD patients was significantly higher among those older than 65 years and with a BMI <25 and DM.

Circulating bilirubin concentrations are primarily derived from hemoglobin of aged or damaged red blood cells. Corresponding, in this present study, it was observed that patients without liver disease in DBIL T3 or T2 had significantly higher concentrations of hemoglobin in contrast to those in T1 (*p* = 0.001). A review shows that increasing plasma bilirubin, acting as an antioxidant and metabolic hormone, can drive metabolic adaptions that improve deleterious outcomes of weight gain and obesity, such as inflammation, T2DM, and cardiovascular diseases (19). Previous studies have proposed a link between bilirubin and AF. However, a recent study conducted on Chinese elderly individuals with CAD and DM demonstrated that DBIL was not a predictor of AF (20). Additionally, a cross-sectional study conducted in patients with paroxysmal atrial fibrillation (pAF) suggested that patients with pAF had significantly higher levels of TBIL and indirect bilirubin, but not DBIL (21). Nevertheless, another study conducted in patients with nonvalvular chronic AF without any other cardiovascular disease revealed that the levels of total, direct and indirect serum bilirubin were significantly lower among the patients with AF compared to controls (18). The reports were inconsistent and controversial. Our study showed that patients with AF had significantly higher levels of both TBIL and DBIL compared to those without AF (*p* = 0.003 and *p* < 0.001, respectively). However, there was no significant difference in liver and kidney function between the two groups. Additionally, univariate and multivariate analysis demonstrated that DBIL was significantly associated with AF in patients with CAD. These findings suggest that DBIL could be a noteworthy marker of AF. Differences in study populations may cause inconsistent results. Moreover, the AF in our study was defined as paroxysmal AF, persistent AF, and permanent AF. Further stratified analysis will be used to evaluate these findings in future studies.

The underling mechanism that may explain the positive association between DBIL and AF have been suggested in several studies, but the precious pathways remain unclear. For decades, both TC and LDL-C have been considered causal factors for atherosclerotic cardiovascular disease. Traditional cardiovascular risk factors, including age, sex, DM, hypertension, for incident AF have been described in early studies (21, 22). Based on the above information, the association between dyslipidemia and atrial fibrillation (AF) has been evaluated. Current studies have indicated that elevated TC and LDL-C levels are inversely associated with incident AF (23, 24). The mechanism underpin the interesting inverse association may be related to the stabilizing effect of cholesterol on myocardial membranes (25–27). Consistently, logistic regression analysis in our study described the role of LDL-C in reduced AF risk. It is also worth noting that DBIL are negatively correlated with TC and LDL-C (28). Similar results were obtained in our study. And analysis revealed that DBIL was inversely associated with TC, TG, LDL-C, and ApoB. Bilirubin might induce hepatic fat utilization and reduce *de novo* lipogenesis, thus lowering lipid levels (29). This may partly explain why atrial fibrillation occurs more frequently in these patients.

Furthermore, as an independent risk factor, DBIL-induced AF may be associated with an inflammatory response. Heme oxygenase-1 (HO-1) and bilirubin IXalpha are predominantly accumulated in the perinuclear regions of foam cells (30). Additionally, foam cells are known to initiate the inflammatory process that may be linked to the pathogenesis of AF (31). Moreover, higher bilirubin levels may reflect increased foam cell presence, which plays a pivotal role in inflammation by regulating the production of inflammatory cytokines and matrix metalloproteinases (32). It was reported that DBIL levels were negatively associated with hsCRP levels (28). Further studies are needed to evaluate the potential role of DBIL in inflammation related to AF, due to the lack of C-reactive protein (CRP), interleukin-6 (IL-6), tumor necrosis factor alpha (TNFα), neutrophil to lymphocyte ratio (NLR), and other inflammatory markers.

The role of DBIL in oxidative stress, which may be associated with AF, is also reported. Defined doses of bilirubin could be considered as mitochondria targeted medication against inflammasome-related diseases (33). However, the molecular pathway(s) connecting reactive oxygen species (ROS) and AF are unknown. The Ca2+/calmodulin-dependent protein kinase II (CaMKII) has recently been suggested to be a ROS activated proarrhythmic signal contributing to AF (34). Oxidant stress levels evaluated by urinary 8-hydroxy-2′-deoxyguanosine (8-OHdG), a biomarker of oxidative DNA damage, and urinary biopyrrin, an oxidative metabolite of bilirubin have been proven to be significantly increased in AF patients (35).

Insulin resistance (IR) has been demonstrated to be an important risk factor for cardiovascular disease. A Prior study reported that bilirubin is associated with insulin resistance. Participants with lower bilirubin had a significantly higher risk of type 2 diabetes (36). Additionally, a cohort study performed by Puerto-Carranza et al. demonstrated that bilirubin can regulate insulin secretion and glucose uptake (37). Furthermore, there was a steep increase in the risk of incident AF associated with relatively low homeostatic model of insulin resistance (HOMA-IR) (38). To date, the TyG index has been shown to be a promising indicator of IR status (39, 40). Consistently, our study found significantly lower TyG levels in the higher DBIL group. These findings may partially elucidate the underling association of DBIL with increased AF risk. Our results could also be attributed to difference in ethnicity and sample size. Further studies are needed to investigate whether this mechanism palys an important role in atrial remodeling and the development of AF.

This study also showed that male gender was inversely correlated with the risk of AF, while older age was identified as an independent risk factor. These findings are consistent with previous studies (17, 28). Previous studies have demonstrated that old age is one of the most recognized risk factors for new onset-atrial fibrillation. Old age has been shown to induce structural and electrical atrial remodeling which increase the risk of developing atrial fibrillation. The prevalence of AF increases dramatically with advancing age (41, 42). Hence, the average age of individuals with AF is significantly higher than that in those without AF. After adjustment for confounding factors, logistic regression analysis revealed that higher age was a significant risk factor for AF. Furthermore, subgroup analysis revealed that the association of higher DBIL levels with the risk of AF was stronger in CAD patients older than 65 years. Prior studies have provided evidence that higher BMI causally increased the risk of AF (43, 44). However, the positive association was not observed in our study. Further stratified analysis suggested that the higher baseline DBIL level was associated with AF risk in individuals with BMI < 25, but not in those with BMI ≥ 25. Selection bias may results in this difference. In addition, Oka et al. (45) found that low BMI and female sex could predict reduced post-ablation left atrial emptying fraction, which was associated with recurrence of AF after ablation. Another possible explanation is that low BMI is a significant predictor of non-pulmonary vein (PV) foci, which plays an important role in the recurrence of AF (46). Prospective studies should be conducted to explore these results further. Prior studies have demonstrated that DM is one of the most common risk factors for the development of AF due to atrial structural and electrical remodeling. Consistent with previous studies (47, 48), a significant association between higher DBIL and AF risk was observed in DM patients. The results of this study suggest that we should pay attention to the DBIL levels of elderly CAD patients with lower BMI and DM in order to detect AF early.

There are several limitations in our study that should be noted. Firstly, this was a cross-sectional study conducted at a single center, and there was no patient follow-up. The results may be prone to selection or information bias. Secondly, the diagnosis of AF based on 12-lead electrocardiogram (ECG) or 24-h Holter may inevitably underestimate rates of AF. Thirdly, the biomarkers of inflammatory including high-sensitivity C-reactive protein and B-type natriuretic peptide, as well as left atrial diameter which need to be considered were not presented and studied. Finally, since AF can occur both paroxysmal and non-paroxysmal, the results should be assessed separately for each category in future studies.

## Conclusion

In conclusion, this present study found a robust nonlinear correlation between serum DBIL levels and AF in patients with CAD. This association was more significant in DM patients and those aged ≥65 years, as well as BMI <25 patients. These findings may help identify CAD patients at high risk of AF by measuring the DBIL levels. In the future, we look forward to design a multicenter study with a larger sample size and long-term follow-up to explore the relationship between DBIL and AF. In addition, DBIL may be a potential target for therapeutic intervention to reduce the risk of AF confirmed by future studies. Further studies are necessary to investigate the role of DBIL in reducing AF risk.

## Data Availability

The raw data supporting the conclusions of this article will be made available by the authors, without undue reservation.

## Glossary

CAD: Coronary artery disease
AF: Atrial fibrillation
DM: Diabetes mellitus
BMI: Body mass index
TBIL: Total bilirubin
DBIL: Direct bilirubin
ALT: Alanine aminotransferase
AST: Aspartate aminotransferase
BUN: Urea nitrogen
Cr: Creatinine
UA: Uric acid
FBG: Fasting blood glucose
TC: Total cholesterol
TG: Triglyceride
HDL-C: High-density lipoprotein cholesterol
LDL-C: Low-density lipoprotein cholesterol
Apo A-1: Apolipoprotein A-1
Apo B: Apolipoprotein B
TyG: Triglyceride-glucose
RCS: Restricted cubic spline

## References

[1] Benjamin EJ Muntner P Alonso A Bittencourt MS Callaway CW Carson AP. Association council on epidemiology and prevention statistics committee and stroke statistics subcommittee. Heart disease and stroke Statistics-2019 update: a report from the American Heart Association. Circulation. (2019) 139:e56–e528. doi: 10.1161/CIR.0000000000000659, PMID: 30700139

[2] January CT Wann LS Alpert JS Calkins H Cigarroa JE Cleveland JC Jr. AHA/ACC/HRS guideline for the management of patients with atrial fibrillation: a report of the American College of Cardiology/American Heart Association task force on practice guidelines and the Heart Rhythm Society. J Am Coll Cardiol. (2014) 130:e1–e76. doi: 10.1161/CIR.0000000000000041, PMID: 24685669

[3] Sygitowicz G Maciejak-Jastrzębska A Sitkiewicz D. A review of the molecular mechanisms underlying cardiac fibrosis and atrial fibrillation. J Clin Med. (2021) 10:4430. doi: 10.3390/jcm10194430, PMID: 34640448 PMC8509789

[4] Lip GY Beevers DG. ABC of atrial fibrillation. History, epidemiology, and importance of atrial fibrillation. BMJ. (1995) 311:1361–3. doi: 10.1136/bmj.311.7016.1361, PMID: 7496293 PMC2551280

[5] Hohnloser SH Crijns HJ van Eickels M Gaudin C Page RL Torp-Pedersen C. Effect of dronedarone on cardiovascular events in atrial fibrillation. N Engl J Med. (2009) 360:668–78. doi: 10.1056/NEJMoa0803778, PMID: 19213680

[6] Nguyen BO Weberndorfer V Crijns HJ Geelhoed B Ten Cate H Spronk H. Prevalence and determinants of atrial fibrillation progression in paroxysmal atrial fibrillation. Heart. (2022) 109:186–94. doi: 10.1136/heartjnl-2022-321027, PMID: 35858774 PMC9872250

[7] Olsen FJ Johansen ND Skaarup KG Lassen MCH Ravnkilde K Schnohr P. Changes in left atrial structure and function over a decade in the general population. Eur Heart J Cardiovasc Imaging. (2021) 23:124–36. doi: 10.1093/ehjci/jeab173, PMID: 34468711

[8] Papazoglou AS Kartas A Moysidis DV Tsagkaris C Papadakos SP Bekiaridou A. Glycemic control and atrial fibrillation: an intricate relationship, yet under investigation. Cardiovasc Diabetol. (2022) 21:39. doi: 10.1186/s12933-022-01473-0, PMID: 35287684 PMC8922816

[9] Yang Y Wang D Zhang C Yang W Li C Gao Z. Piezo 1 mediates endothelial atherogenic inflammatory responses via regulation of YAP/TAZ activation. Hum Cell. (2022) 35:51–62. doi: 10.1007/s13577-021-00600-5, PMID: 34606042

[10] Bobescu E Marceanu LG Dima L Balan A Strempel CG Covaciu A. Trimetazidine therapy in coronary artery disease: the impact on oxidative stress, inflammation, endothelial dysfunction, and long-term prognosis. Am J Ther. (2021) 28:e540–7. doi: 10.1097/MJT.0000000000001430, PMID: 34321406

[11] Shao C Wang J Tian J Tang YD. Coronary artery disease: from mechanism to clinical practice. Adv Exp Med Biol. (2020) 1177:1–36. doi: 10.1007/978-981-15-2517-9_1 32246442

[12] Yu FF Yuan Y Ao Y Hua L Wang W Cao Y. A new product of bilirubin degradation by H2O2 and its formation in activated neutrophils and in an inflammatory mouse model. Biomol Ther. (2022) 12:1237. doi: 10.3390/biom12091237, PMID: 36139076 PMC9496627

[13] Zhu Y Wu X Liu H Niu Z Zhao J Wang F. Employing biochemical biomarkers for building decision tree models to predict bipolar disorder from major depressive disorder. J Affect Disord. (2022) 308:190–8. doi: 10.1016/j.jad.2022.03.080, PMID: 35439462

[14] Wang J Xie L Lyu P Zhou F Cai HL Qi RX. Short-term prognostic efficacy of mGPS and LCS in patients With acute Heart failure. Front Cardiovasc Med. (2022) 9:944424. doi: 10.3389/fcvm.2022.944424, PMID: 35865381 PMC9295910

[15] Turkkolu ST Selçuk E Köksal C. Biochemical predictors of postoperative atrial fibrillation following cardiac surgery. BMC Cardiovasc Disord. (2021) 21:167. doi: 10.1186/s12872-021-01981-z, PMID: 33836659 PMC8033715

[16] Sun D Li W Zheng W Tan J Zhang G. Direct bilirubin level is an independent risk factor for atrial fibrillation in thyrotoxic patients receiving radioactive iodine therapy. Nucl Med Commun. (2019) 40:1289–94. doi: 10.1097/MNM.0000000000001107, PMID: 31725052

[17] Sun W Li H Wang Z Wu Y Du J. Clinical and laboratory biomarkers in paroxysmal atrial fibrillation: a single center cross-sectional study. Contrast Media Mol Imaging. (2022) 2022:7012377. doi: 10.1155/2022/7012377, PMID: 35845733 PMC9259273

[18] Demir M Demir C Uyan U Melek M. The relationship between serum bilirubin concentration and atrial fibrillation. Cardiol Res. (2013) 4:186–91. doi: 10.4021/cr299w, PMID: 28352443 PMC5358307

[19] Thomas DT DelCimmuto NR Flack KD Stec DE Hinds TD Jr. Reactive oxygen species (ROS) and antioxidants as Immunomodulators in exercise: implications for Heme oxygenase and bilirubin. Antioxidants (Basel). (2022) 11:179. doi: 10.3390/antiox11020179, PMID: 35204062 PMC8868548

[20] Xu Q Peng Y Tan J Zhao W Yang M Tian J. Prediction of atrial fibrillation in hospitalized elderly patients With coronary Heart disease and type 2 diabetes mellitus using machine learning: a multicenter retrospective study. Front Public Health. (2022) 10:842104. doi: 10.3389/fpubh.2022.842104, PMID: 35309227 PMC8931193

[21] Chyou JY Hunter TD Mollenkopf SA Turakhia MP Reynolds MR. Individual and combined risk factors for incident atrial fibrillation and incident stroke: an analysis of 3 million at-risk US patients. J Am Heart Assoc. (2015) 4:e001723. doi: 10.1161/JAHA.114.001723, PMID: 26206736 PMC4608064

[22] Brunner KJ Bunch TJ Mullin CM May HT Bair TL Elliot DW. Clinical predictors of risk for atrial fibrillation: implications for diagnosis and monitoring. Mayo Clin Proc. (2014) 89:1498–505. doi: 10.1016/j.mayocp.2014.08.016, PMID: 25444486

[23] Li X Gao L Wang Z Guan B Guan X Wang B. Lipid profile and incidence of atrial fibrillation: a prospective cohort study in China. Clin Cardiol. (2018) 41:314–20. doi: 10.1002/clc.22864, PMID: 29575115 PMC6490045

[24] Lopez FL Agarwal SK Maclehose RF Soliman EZ Sharrett AR Huxley RR. Blood lipid levels, lipid-lowering medications, and the incidence of atrial fibrillation: the atherosclerosis risk in communities study. Circ Arrhythm Electrophysiol. (2012) 5:155–62. doi: 10.1161/CIRCEP.111.966804, PMID: 22227953 PMC3290134

[25] Dart C. Lipid microdomains and the regulation of ion channel function. J Physiol. (2010) 588:3169–78. doi: 10.1113/jphysiol.2010.191585, PMID: 20519314 PMC2976012

[26] Goonasekara CL Balse E Hatem S Steele DF Fedida D. Cholesterol and cardiac arrhythmias. Expert Rev Cardiovasc Ther. (2010) 8:965–79. doi: 10.1586/erc.10.79, PMID: 20602558

[27] Abi-Char J Maguy A Coulombe A Balse E Ratajczak P Samuel JL. Membrane cholesterol modulates Kv1.5 potassium channel distribution and function in rat cardiomyocytes. J Physiol. (2007) 582:1205–17. doi: 10.1113/jphysiol.2007.134809, PMID: 17525113 PMC2075263

[28] Fu J Wang Q Zhang L Liu J Wang G. Serum bilirubin level is increased in metabolically healthy obesity. Front Endocrinol. (2022) 12:792795. doi: 10.3389/fendo.2021.792795, PMID: 35432184 PMC9005889

[29] Hinds TD Jr Creeden JF Gordon DM Stec DF Donald MC Stec DE. Bilirubin nanoparticles reduce diet-induced hepatic steatosis, improve fat utilization, and increase plasma β-Hydroxybutyrate. Front Pharmacol. (2020) 11:594574. doi: 10.3389/fphar.2020.594574, PMID: 33390979 PMC7775678

[30] Nakayama M Takahashi K Komaru T Fukuchi M Shioiri H Ki S. Increased expression of heme oxygenase-1 and bilirubin accumulation in foam cells of rabbit atherosclerotic lesions. Arterioscler Thromb Vasc Biol. (2001) 21:1373–7. doi: 10.1161/hq0801.093592, PMID: 11498468

[31] Wang D Yang Y Lei Y Tzvetkov NT Liu X Yeung AWK. Targeting foam cell formation in atherosclerosis: therapeutic potential of natural products. Pharmacol Rev. (2019) 71:596–670. doi: 10.1124/pr.118.017178, PMID: 31554644

[32] Naito M. Macrophage differentiation and function in health and disease. Pathol Int. (2008) 58:143–55. doi: 10.1111/j.1440-1827.2007.02203.x, PMID: 18251777

[33] Li Y Sheng H Yan Z Guan B Qiang S Qian J. Bilirubin stabilizes the mitochondrial membranes during NLRP3 inflammasome activation. Biochem Pharmacol. (2022) 203:115204. doi: 10.1016/j.bcp.2022.115204, PMID: 35944727

[34] Purohit A Rokita AG Guan X Chen B Koval OM Voigt N. Oxidized ca (2+)/calmodulin-dependent protein kinase II triggers atrial fibrillation. Circulation. (2013) 128:1748–57. doi: 10.1161/CIRCULATIONAHA.113.003313, PMID: 24030498 PMC3876034

[35] Toyama K Yamabe H Uemura T Nagayoshi Y Morihisa K Koyama J. Analysis of oxidative stress expressed by urinary level of 8-hydroxy-2′-deoxyguanosine and biopyrrin in atrial fibrillation: effect of sinus rhythm restoration. Int J Cardiol. (2013) 168:80–5. doi: 10.1016/j.ijcard.2012.09.068, PMID: 23040081

[36] Wei Y Liu C Lai F Dong S Chen H Chen L. Associations between serum total bilirubin, obesity and type 2 diabetes. Diabetol Metab Syndr. (2021) 13:143. doi: 10.1186/s13098-021-00762-0, PMID: 34876211 PMC8650363

[37] Puerto-Carranza E Nuevo-Casals S Roca-Portella B Mas-Parés B Gómez-Vilarrubla A Carreras-Badosa G. Total bilirubin and bilirubin-to-triglycerides ratio predict changes in glycated hemoglobin in healthy children. Front Endocrinol. (2023) 14:1303597. doi: 10.3389/fendo.2023.1303597, PMID: 38107514 PMC10722262

[38] Polovina M Krljanac G Ašanin M Seferović PM. Crouching tiger, hidden dragon: insulin resistance and the risk of atrial fibrillation. Eur J Prev Cardiol. (2020) 27:1931–3. doi: 10.1177/2047487320912626, PMID: 32237896

[39] Luo E Wang D Yan G Qiao Y Liu B Hou J. High triglyceride-glucose index is associated with poor prognosis in patients with acute ST-elevation myocardial infarction after percutaneous coronary intervention. Cardiovasc Diabetol. (2019) 18:150. doi: 10.1186/s12933-019-0957-3, PMID: 31722708 PMC6852896

[40] Du T Yuan G Zhang M Zhou X Sun X Yu X. Clinical usefulness of lipid ratios, visceral adiposity indicators, and the triglycerides and glucose index as risk markers of insulin resistance. Cardiovasc Diabetol. (2014) 13:146. doi: 10.1186/s12933-014-0146-3, PMID: 25326814 PMC4209231

[41] Bizhanov KA Аbzaliyev KB Baimbetov AK Sarsenbayeva AB Lyan E. Atrial fibrillation: epidemiology, pathophysiology, and clinical complications (literature review). J Cardiovasc Electrophysiol. (2023) 34:153–65. doi: 10.1111/jce.15759, PMID: 36434795

[42] Gao P Gao X Xie B Tse G Liu T. Aging and atrial fibrillation: a vicious circle. Int J Cardiol. (2024) 395:131445. doi: 10.1016/j.ijcard.2023.131445, PMID: 37848123

[43] Zhao M Du W Zhao Q Chen Y Li B Xie Z. Transition of metabolic phenotypes and risk of atrial fibrillation according to BMI: Kailuan study. Front Cardiovasc Med. (2022) 9:888062. doi: 10.3389/fcvm.2022.888062, PMID: 35837597 PMC9274110

[44] Chen W Yao D Yan H Wang M Pan Y. Genetically predicted childhood obesity and adult atrial fibrillation: a mendelian randomization study. Nutr Metab Cardiovasc Dis. (2022) 32:1019–26. doi: 10.1016/j.numecd.2021.12.001, PMID: 35086764

[45] Oka T Koyama Y Tanaka K Hirao Y Tanaka N Okada M. Post-ablation left atrial function impacts long-term recurrence of atrial fibrillation after ablation. Heart Vessel. (2022) 37:315–26. doi: 10.1007/s00380-021-01915-x, PMID: 34342674

[46] Inamura Y Nitta J Inaba O Sato A Takamiya T Murata K. Presence of non-pulmonary vein foci in patients with atrial fibrillation undergoing standard ablation of pulmonary vein isolation: clinical characteristics and long-term ablation outcome. Int J Cardiol Heart Vasc. (2021) 32:100717. doi: 10.1016/j.ijcha.2021.100717, PMID: 33532545 PMC7822950

[47] Karam BS Chavez-Moreno A Koh W Akar JG Akar FG. Oxidative stress and inflammation as central mediators of atrial fibrillation in obesity and diabetes. Cardiovasc Diabetol. (2017) 16:120. doi: 10.1186/s12933-017-0604-9, PMID: 28962617 PMC5622555

[48] Wang A Green JB Halperin JL Piccini JP Sr. Atrial fibrillation and diabetes mellitus: JACC review topic of the week. J Am Coll Cardiol. (2019) 74:1107–15. doi: 10.1016/j.jacc.2019.07.020, PMID: 31439220

